# Livestock ownership, household food security and childhood anaemia in rural Ghana

**DOI:** 10.1371/journal.pone.0219310

**Published:** 2019-07-24

**Authors:** Aaron Kobina Christian, Mark L. Wilson, Richmond N. O. Aryeetey, Andrew D. Jones

**Affiliations:** 1 Regional Institute for Population Studies, University of Ghana, Legon, Accra, Ghana; 2 Department of Epidemiology, School of Public Health, University of Michigan, Ann Arbor, Michigan, United States of America; 3 Department of Population, Family and Reproductive Health, School of Public Health, University of Ghana, Legon, Accra, Ghana; 4 Department of Nutritional Sciences, School of Public Health, University of Michigan, Ann Arbor, Michigan, United States of America; University of Dhaka, BANGLADESH

## Abstract

The mechanisms through which livestock ownership is associated with childhood anaemia are contested. Using a cross-sectional, community-based survey of 300 households in southern Ghana, we determined the associations of household livestock ownership with anaemia among children aged 2–5 years. Potential mediating effects of animal-source food (ASF) consumption, microbial infections, and household food security were investigated. Data on each child's anaemia, malaria, and intestinal infections were collected for a subset of 221 households. Anaemia was defined as a haemoglobin (Hb) concentration <110 g/L. ASF consumption was measured as a count of the number of different ASF types consumed by each child in the week prior to the interview. Household food security was measured with a 15-item, pre-tested tool adapted from the USDA Household Food Security Core Module. The number of sheep and goats in aggregate was associated with higher odds of a child being anaemic (aOR (95% CI) = 1.10 (1.03, 1.17)). Households owning more free-range poultry had greater diversity of consumed ASFs among children (Coef. (95% C) = 0.02 (0.01, 0.03)). Owning more pigs was associated with higher odds that a household was food secure (1.05 (0.99, 1.12). We found no evidence that the child's ASF consumption mediated the association of livestock ownership with child anaemia, however,household food security mediated the association between household pig ownership and child anaemia. Overall, household ownership of livestock was associated with higher ASF consumption among children and improved household-level food security, yet also a higher odd of anaemia among those young children. The mechanisms leading to these seemingly counterintuitive relationships require further investigation.

## Introduction

Anaemia remains a major public health problem, particularly in sub-Saharan Africa [1]. In addition to being an important cause of mortality in children admitted to hospitals, anaemia can lead to impaired cognitive function, poor school performance, and poor growth in children [2, 3]. Diverse factors contribute to anaemia in children, including dietary micronutrient deficiencies of micronutrients and infectious diseases (e.g., malaria, intestinal helminths, and diarrhoea), suggesting that a multi-sectoral strategy is needed to address the problem [4].

Nutrition-sensitive agriculture interventions such as the promotion of animal husbandry and consumption of animal-source foods (ASFs) have the potential to improve anaemia and overall growth among rural children [5, 6]. Indeed, household ownership of livestock, particularly among rural households, is also associated with greater food security [7]. Such enhanced food security is linked with improved intake of micronutrients, including iron, which is essential for preventing iron deficiency anaemia [8]. Ironically, the presence of household animals may also increase the likelihood of certain infections that elevate anaemia risk, particularly among young children [9]. The potential linkages among household livestock ownership, household food security, and anaemia in children are complex and potentially contradictory.

**Fig 1:**
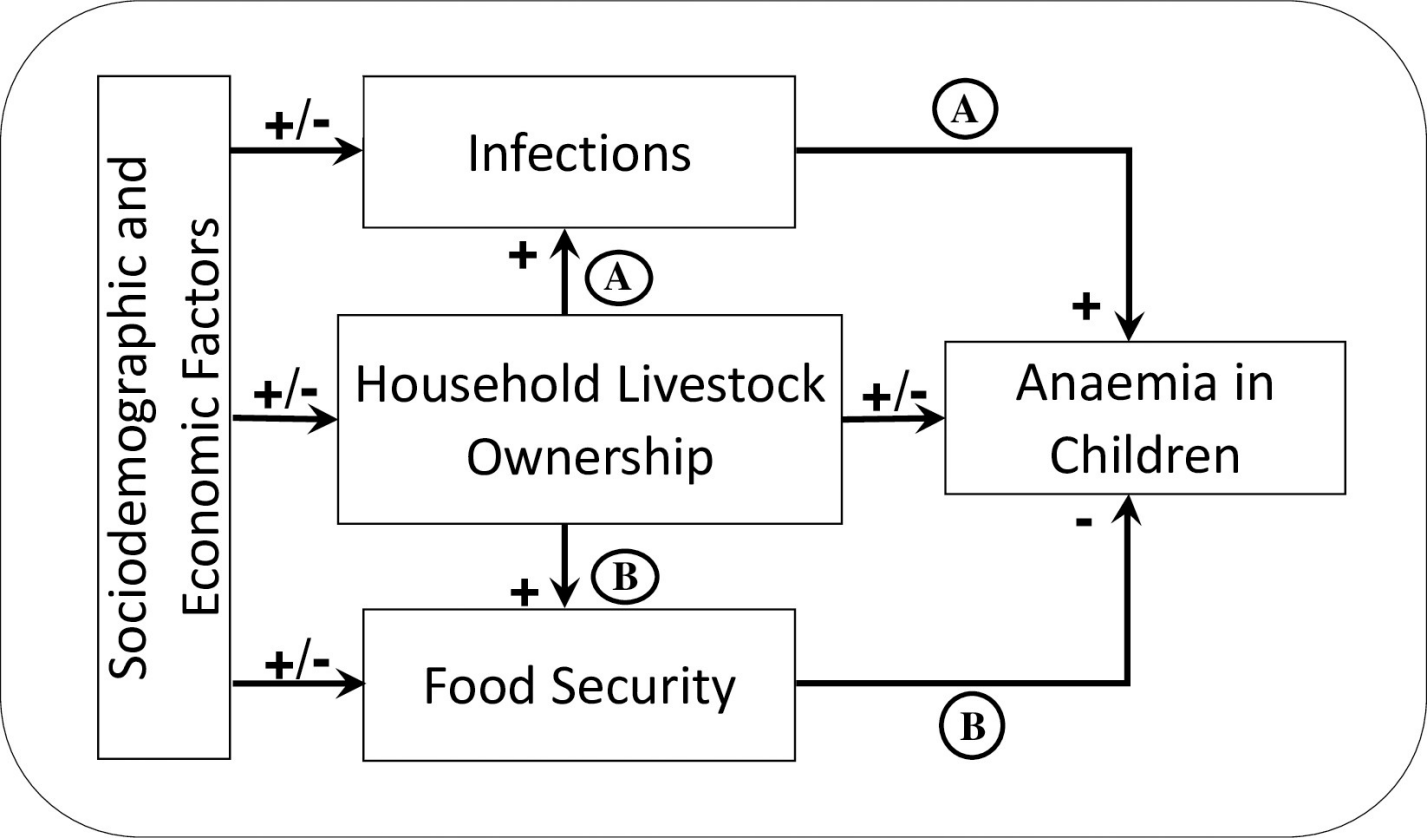
Relationship between household livestock ownership and anaemia in children.

Sociodemographic and economic characteristics of households, for example, the water, sanitation, and hygiene environment of households have been shown to influence the exposure of children to infections [10], while household land ownership and higher wealth positively impact food security [11]. Fig 1 illustrates two possible pathways from household livestock ownership to children’s anaemia status. Pathway A suggests that household ownership of livestock may increase exposure to infections, which in turn may increase anaemia in children via inflammation. Pathway B indicates that household livestock ownership may improve household food security, which in turn may decrease anaemia through improved dietary intake.

The above notwithstanding, few studies have explored the relationship between livestock ownership, infections, and anaemia in concert with household food security [12, 13]. Two recent studies by Kaur et al. (2017) and Jones et al. (2018), have explored the nexus between household livestock ownership, infections, and child health outcomes [9]. Both studies analysed Demographic and Health Survey data, with the former concluding that in sub-Saharan African countries, livestock may simultaneously protect against child stunting but increase the risk of all-cause mortality in children [14]. The latter analysis, focused on Ghana, showed that livestock ownership had a limited association with consumption of ASFs, but was significantly related to greater risk of children being anaemic. Neither study examined the direct and indirect effects of livestock ownership on child anaemia status through infections or household food security. Therefore, in this study, we aimed to determine the association between household livestock ownership and anaemia among children, and the contribution of household food security and infection as potential mechanisms mediating this association.

## Methods

### Data and study design

We conducted a cross-sectional study in the Upper Manya Krobo District of the Eastern region of Ghana during January and February 2018. This district lies within the semi-deciduous forest and savanna agroecological zones of Ghana. Most residents (87.2%) of the district live in rural communities, and the majority of households (82.5%) are engaged in agriculture-related activities [15]. Factors such as increased exposure to intestinal infestation, malaria and poor dietary choices contribute to the high prevalence of childhood anaemia in rural communities such as the current study site.

More than half (56.6%) of the children in this district have been shown to be anaemic with 11.0% being stunted and/or underweight [16, 17]. Earlier studies in this district have indicated that iron was the least adequate micronutrient consumed among infants in this area (RIING Project, unpublished results).

The following formula was used to calculate the sample size for the study:

$$N=Z^{2}P\left(1-P\right)/d2,$$

where N is the sample size, Z is the statistic corresponding to the level of confidence, P is the expected prevalence and d is precision (corresponding to effect size) [18]. With an estimated prevalence (p) of 25.0% of 6–59 months children being moderately anaemic in the study area (16), a level of confidence level of 95%, and a precision (d) of 0.05, we calculated that a sample size of 288 children was needed. The sample was rounded up to 300 households. Target households were those with a child aged between two and five years.

Based on available resources, three communities were randomly selected from a sample frame of all villages and communities in the district. A census of households with 2-to 5-year-old children in selected communities was conducted, and index caregiver-child dyads were identified in each household. The number of households selected in each community was based on probability proportional to the number of children living in each community.

The index caregiver in each household was considered as the person living with a 2-to 5-year-old child who had primary responsibility for feeding the child. Children with any visible illness and/or congenital abnormalities, and children with known positive sickle cell status were excluded. For households with more than one eligible child, the youngest child was chosen for inclusion in the study. Three hundred households with at least one healthy child aged 2–5 years were randomly selected. A multi-module, interviewer-administered, questionnaire survey was conducted through one-on-one interviews by trained enumerators who were experienced in field data collection and the practice of responsible and ethical research data collection. Although we interviewed 300 for the survey, we were only able to collect biological samples for 221 children because of a time lag of one to three days between the survey interviews and blood, stool and urine sample collection. In total, 79 households were unavailable for sample data collection following three repeat visits to the households in an attempt to collect these data.

The study was approved by the Institutional Review Board (IRB) of the Noguchi Memorial Institute for Medical Research, University of Ghana, Legon and all participants provided written informed consent. This research was also embedded within the Post-Doctoral and Research Trainee NEtwoRk-Investing in Innovation (PARTNER II) research program for post-doctoral fellows at the University of Michigan. Thus, the study protocol was also approved by the University of Michigan Health Sciences and Behavioral Sciences Institutional Review Board.

#### Measurement of variables

Child haemoglobin (Hb) concentrations were assessed from capillary blood samples using a HemoCue photometer system (URIT Medical Electronic Group, Guilin, Guangxi, China) [19]. Children with Hb <11.0 g/dL were defined as anaemic [20]. Ownership of any livestock and the number of free-range poultry, corral chickens, sheep, goats, and pigs owned by households served as the primary independent variables. Guided by existing evidence on factors that influence the anaemia status of children, we controlled for selected sociodemographic characteristics [9, 21, 22]. Using a food frequency questionnaire, caregivers were asked about children’s dietary intake in the week prior to the interview. ASF consumption by children was measured as a count of the number of different ASFs consumed by the child in the week prior to the interview. Possible categories of ASF consumed were (a) any meat (e.g., beef, pork, lamb, goat, chicken, or duck); (b) liver, kidney, heart, or other organ meats; (c) fresh or dried fish or shellfish; (d) eggs; and (e) milk, cheese, or other food made from milk [23].

We also included child malaria status as a covariate. Malaria status was determined using a standard malaria RDT kit (Wondfo One Step Malaria P.f/P.v Whole Blood Test) to qualitatively identify the presence of *Plasmodium* species (Wondfo-POCT, China). We further recorded caregiver-reported child diarrhoea or fever during two weeks prior to the interview, as well as caregiver-reported child- and household-level water, sanitation, and hygiene (WASH) practices and characteristics (caregiver-reported open defecation in the household compound and child hand-washing with soap and water) [24]. The presence of garbage or a garbage collection point <10 meters from the house, and the distance to any open water body, drainage system and/or gutters were also assessed.

We also asked whether, during the past six months, the child had been treated for an intestinal infection or given vitamin A supplementation. Household food security was measured with a 15-item, pre-tested tool adapted from the USDA Household Food Security Core Module [25]. Positive responses to the 15 dichotomous food security questions were summed to give a total food insecurity score that was used to categorize households as food secure (0 positive responses), mildly food insecure (1–5 positive responses), moderately food insecure (6–10 positive responses), or severely food insecure (11–15 positive responses). We generated a household wealth index from a principal component analysis of household assets. Derived wealth scores were extracted from the first component and categorized into tertiles (low, medium, and high).

Caregivers were provided with sterile containers one day prior to the community survey for the collection of their study child’s stool and urine samples. They were given verbal instructions about how to aseptically place at least 2–3 scoops of faeces (3 mL) and a quantity of urine into the containers, then tightly close the containers. The sample containers were gathered the next morning by field personnel. A portion of the collected stool sample was aliquoted with 10% formalin preservative for analysis. The rest of each sample was frozen (-4°C) and transported for analysis at the Noguchi Memorial Institute for Medical Research (NMIMR). Kato-Katz analysis was done to detect infection with hookworm, *Ascaris* sp., *Trichiura trichiura*, *Hymenolepis nana*, *Shistosoma mansoni*, and *Taenia* sp.[26]. Collected urine samples were also tested for *S*. *heamatobium* and *S*. *mansoni* [27].

#### Statistical analysis

All analyses were done using the Stata statistical software package version 14.2 (2017; StataCorp, College Station, TX, USA). Two‐sided Student's t-test statistics and Pearson's chi‐squared test statistics were conducted to test for differences in means and proportions, respectively, between households with and without livestock.

Logistic regression was used to model the associations of household livestock ownership and ownership of specific livestock types with child anaemia (modelled as a binary outcome). As intestinal infections and malaria were hypothesized to increase inflammation risk that may, in turn, contribute to anaemia, analyses controlled for malaria, intestinal infections, and household-level environment characteristics such as proximity to water sources that may be linked to infection. Adjusted models controlled for additional household, caregiver, and child-level characteristics known and hypothesized to influenced anaemia. These included householdwealth, sex of head of household, household food security, proximity of household to a garbage collection point, and water body, and type of drainage system/gutter belonging to the household, formal education status and marital status of the caregiver, and the age and sex of the child, recent child consumption of ASFs, presence of fever, malaria, and/or intestinal parasites among the child, whether the child had taken medication against worm infestations, washes hands with soap, and/or openly defecates.

We further assessed the contribution of infection, child ASF consumption, and household food security as potential mediating mechanisms of the association of household livestock ownership with anaemia. Logistic regression was also used to model the association of household livestock ownership and ownership of specific livestock types with household food security (modelled as a binary outcome).

Linear regression was employed to examine the associations of household livestock ownership and ownership of specific livestock types with children’s ASF diversity. Consumption from six categories of ASFs was assessed including:(a) any meat(e.g., beef, pork, lamb, goat, chicken, or duck); (b) liver, kidney, heart, or other organ meats; (c) fresh or dried fish or shellfish; (d) poultry meat, (e) eggs; and (f) milk, cheese, or other food made from milk. ASF diversity was modelled as a continuous variable ranging from 0 to 6 with 0 indicating no recent consumption of ASFs, and to 6 indicating the child consumed from all categories of ASF within the past week prior to the survey. The three main explanatory variables explored were the number of free-range poultry, the number of sheep and goats in aggregate and the number of pigs. Other covariates were household wealth, household size, the sex of the household head, and caregiver’s marital status and educational level.

## Results

In total, 300 households participated in the study, with data on anaemia, malaria, and other intestinal infections collected from the study child in each of 221 households. Approximately one-third of the households (34%) were food secure while 33%, 24% and 9% were mildly, moderately, and severely food insecure, respectively.

About a third of households (32.7%) owned free-range chickens. Only 1.7% of households owned other birds (i.e., duck, turkey, and corralled chicken), and approximately one-fifth of households (21%) owned goats. Sheep and pigs were owned by 9.3% and 2.7% of the households, respectively. Overall 44.7% (134 households) owned a type of livestock. The mean age of primary caregivers was 30.9 y (8.9) SD and that of index children was 40.2 months (11.4). The mean ASF diversity for children was 1.2 (1.2).

The prevalence of anaemia, malaria, and intestinal infections were 46.3%, 23.4% and 5.0%, respectively. Table 1 shows selected household, caregiver- and child-level characteristics by household livestock ownership Bivariate analysis showed a significantly higher proportion of children with infections in households without livestock than their counterparts in households with livestock. The mean number of persons in households without livestock was significantly less than households without livestock (mean± SD; 6.1±2.1 vs.7.4 ± 2.9 respectively). There were no other significant differences in other selected household, caregiver-, or child-level characteristics by households with and without livestock.

**Table 1:** Household, caregiver- and child-level characteristics, by household livestock ownership.

			Households without livestock (N = 166)		Household with livestock N = 134		p-value
Household wealth^1^							0.685
		Low	35.0	(58)	31.3	(42)	
		Medium	33.7	(56)	32.8	(44)	
		High	31.3	(52)	35.8	(48)	
Household food security							0.381
		Food secure	35.5	(59)	32.1	(43)	
		Mildly food insecure	31.3	(52)	34.3	(46)	
		Moderately food insecure	21.7	(36)	26.9	(36)	
		Severely food insecure	11.5	(19)	6.7	(9)	
Household members (n)			6.1 ± 2.7		7.4 ± 2.9		<0.001
**Caregiver**							
Caregiver formal education							0.880
		No education	32.5	(54)	32.1	(43)	
		Preschool/Primary	38.0	(63)	35.8	(48)	
		Secondary and above	29.5	(49)	32.1	(43)	
Marital status							0.434
		Married/cohabit	84.9	(141)	88.1	(118)	
		Unmarried	15.1	(25)	11.9	(16)	
Caregiver age (yrs)			30.3 ± 8.8		31.5 ± 9.1		0.289
**Children**							
	Age in months		39.5 ±11.5		41.1 ± 11.3		0.220
	ASF diversity		1.2 ± 1.2		1.2 ± 1.1		0.997
	Anaemia status^2^						0.244
		Anaemic	42.6	(49)	50.5	(52)	
		Not anaemic	57.4	(66)	49.5	(51)	
	Intestinal infection^2^^,^^3^						0.051
		Not infected	92.3	(108)	98.1	(101) **	
		Infected	7.7	(9)	1.9	(2)	
	Malaria (*Plasmodium* parasite)						0.292
		Positive	20.2	(23)	26.2	(27)	
		Negative	79.8	(91)	73.8	(76)	

Values represent % (Frequency) or Mean ± Standard deviation

***p≤0.001

** p≤0.05

* p≤0.1

^**1**^Householdwealth is based on tertiles for the first component of a principal component analysis using household assets. N = 300 (n = 134: households with livestock; n = 166: households without livestock).

^2^A sub-sample of 218 children was available for selected biological indicators including anaemia status, malaria status and intestinal infestation.

^3^Intestinal infections include hookworm, Ascaris, T. trichiura, H. nana, S. mansoni, Taenia, S. heamatobium, S. mansoni.

### Livestock ownership association with anaemia in children

Table 2 shows multiple logistic regression analysis for adjusted effects of number of different livestock in a household and livestock ownership on child anaemia. After adjusting for other covariates, household ownership of sheep and goats (i.e., the total numberof sheep and goats owned in aggregate) was associated with higher odds of anaemia in children (aOR (95% CI) = 1.10 (1.03, 1.17).

**Table 2:** Adjusted logistic regression analyses for predictors of childhood anaemia.

			Odds Ratio	Standard Error	P-values	95% Confident Interval	
Livestock							
	Number of free-range poultry		1.05	0.02	0.06	[1.00	1.10]
	Number of sheep & goats		1.10**	0.04	**0.01**	**[1.03**	**1.17]**
	Number of pigs		1.09	0.07	0.18	[0.96	1.22]
	Owns any livestock (ref: No)						
		Yes	0.32**	0.15	**0.02**	**[0.12**	**0.81]**
Household wealth (ref: low)							
	Medium		0.84	0.38	0.70	[0.35	2.03]
	High		1.06	0.49	0.90	[0.42	2.64]
Household size			1.06	0.06	0.34	[0.95	1.18]
Sex of household head (Ref: Male)							
	Female		1.91	0.87	0.16	[0.78	4.67]
Food security (Ref: Food secure)							
	Mildly food insecure		0.45	0.24	0.14	[0.16	1.29]
	Moderately food insecure		0.49	0.27	0.19	[0.17	1.43]
	Severely food insecure		0.40	0.28	0.19	[0.10	1.55]
Household close to a cabbage collection point (ref: No)							
	Yes		1.00	0.38	0.99	[0.48	2.11]
House drainage system/gutter (Ref: no gutter)							
	Close to an open drainage		0.73	0.40	0.57	[0.25	2.14]
	Close to a closed drainage		0.25**	0.12	**<0.05**	**[0.10**	**0.63]**
Close to a waterbody (Ref: not close)							
	Yes		2.17	0.99	0.09	[0.89	5.33]
Caregiver Marital Status (Ref: Single)							
	Married		1.78	0.91	0.26	[0.65	4.86]
Education (Ref: No education)							
	Preschool/Primary		0.84	0.37	0.69	[0.35	1.99]
	Secondary		1.50	0.65	0.36	[0.63	3.52]
Child Sex (Ref: Female)							
	Male		1.52	0.54	0.24	[0.76	3.03]
Child age in months			1.00	0.00	0.29	[1.00	1.00]
Child ASF diversity			0.87	0.13	0.33	[0.65	1.16]
Presence of malaria parasites (Ref: No)							
	Yes		4.19**	1.94	**<0.05**	**[1.69**	**10.37]**
Presence of intestinal parasites (Ref: No)							
	Yes		0.75	0.81	0.79	[0.09	6.21]
Presence of Fever (Ref: No)							
	Yes		0.61	0.22	0.17	[0.30	1.24]
Taken medication against worm infestation (Ref: No)							
	Yes		1.63	0.56	0.15	[0.84	3.18]
Child opening defecate (Ref: No)							
	Yes		0.82	0.40	0.68	[0.31	2.14]
Child washes hands with soap (ref: No)							
	Yes		0.94	0.41	0.89	[0.40	2.21]

**p < .05. Anaemia modelled as a dichotomous variable (Child anaemic = 1, not anaemic = 0)

Household ownership of any livestock was associated with lower odds of anaemia among children (aOR (95% CI) = (0.32 (0.12, 0.81)).

Children in households with closed drainage system/gutters (compared to households without any drainage/gutters) had lower odds of anaemia (0.25, (0.10, 0.63)), children living close to a water body, and who tested positive for malaria had approximately two-fold and four-fold higher odds of anaemia, respectively (2.17, (0.89, 5.33)), and 4.19 (1.69, 10.37)).

### Livestock ownership and association with child consumption of ASFs

Table 3 shows regression analysis for adjusted effects of number of different livestock in a household and livestock ownership on child consumption of animal source foods. In unadjusted multiple linear regression analyses, an increase in the total number of free-range poultry was significantly associated with greater ASF diversity of children (Coef, (95% CI) = 0.02 (0.01, 0.03)). After adjusting for other covariates, an increase in the total number of free-range poultry was still associated with higher ASF diversity (0.02 (0.01, 0.03)), however, ownership of any livestock was associated with decreased consumption of ASF (Table 3).

**Table 3:** Linear regression analyses for the association of household ownership of livestock with children’s consumption of ASF.

		Coefficient.	Standard Error	P-value	95% Conf. Interval	
Livestock						
	Number of free-range poultry	0.02**	0.01	**<0.05**	**[0.01**	**0.03]**
	Number of sheep & goats	0.02	0.02	0.23	[-0.01	0.06]
	Number of pigs	-0.02	0.01	0.12	[-0.05	0.01]
Owns any livestock (ref: No)						
	Yes	-0.36**	0.17	**0.04**	**[-0.70**	**-0.02]**
Household wealth (Ref: Low wealth)						
	Medium	0.56**	0.16	**<0.05**	**[0.25**	**0.88]**
	High	0.63**	0.17	**<0.05**	**[0.30**	**0.96]**
Number of persons		0.02	0.03	0.36	[-0.03	0.08]
Sex of household head (Ref: Male)						
	Female	0.31	0.22	0.17	[-0.13	0.74]
Caregiver Marital Status (Ref: Single)						
	Married	0.57**	0.18	**<0.05**	**[0.22**	**0.93]**
Education (Ref: No education)						
	Preschool/Primary	0.38**	0.16	**0.02**	**[0.06**	**0.70]**
	Secondary	0.41**	0.17	**0.02**	**[0.08**	**0.75]**
Child						
	Sex (Ref: Female)					
	Male	-0.12	0.14	0.39	[-0.40	0.15]
	Age in months	-0.01	0.01	0.30	[-0.02	0.01]

**p < .05. Children’s ASF diversity is represented as continuous outcome i.e. the number of ASF groups a child consumes from (offal’s-liver/kidney/heart/gizzard, beef/goat, poultry, eggs, dairy products and Fish) consumed by children over the past

A higher ASFs diversity was consumed by children from households with high wealth status compared to low wealth (0.63, (0.30, 0.96)), when caregivers had attended secondary school and above (compared to no education) (0.41 (0.08, 0.75)), and caregivers being married (compared to single caregivers) (0.57, (0.22, 0.93)) were also associated with a higher diversity of ASFs consumed by children.

### Livestock ownership and association with household food security

Table 4 shows the association of household ownership of livestock with household food security. After adjusting for household sociodemographic and economic characteristics and other livestock, the number of pigs owned was associated with higher odds of households being food secure respectively (aOR 1.42, 95% CI [0.11, 1.80]). Compared to low wealth households, high wealth households had higher odds of being food secure (aOR 2.82, 95% CI [1.41, 5.62]).

**Table 4:** Adjusted logistic regression analyses for predictors of Household food security.

		Odds Ratio	Standard Error	P-value	[95% Conf. Interval]	
Livestock						
	Number of free-range poultry	1.00	0.02	0.87	[0.97	1.04]
	Number of sheep & goats	1.05	0.03	0.11	[0.99	1.12]
	Number of pigs	1.42**	0.17	**0.01**	**[1.11**	**1.80]**
Owns any livestock (ref: No)						
	Yes	0.52	0.21	0.10	[0.24	1.13]
Household wealth (ref: Low wealth)						
	Medium	1.31	0.45	0.43	[0.67	2.58]
	High	2.82**	0.99	**<0.05**	**[1.41**	**5.62]**
Number of persons		0.96	0.05	0.51	[0.87	1.07]
Sex of household head (ref: Male)						
	Female	0.42	0.20	**0.07**	**[0.16**	**1.09]**
Caregiver Marital Status (ref: Single)						
	Married	1.19	0.53	0.70	[0.50	2.83]
Education (ref: No education)						
	Preschool/Primary	1.38	0.48	0.34	[0.71	2.71]
	Secondary	1.34	0.48	0.42	[0.66	2.72]
Women age in years		0.99	0.02	0.66	[0.96	1.03]

**p < .05. Household food security modelled as a dichotomous variable (Food secure = 1, Food insecure = 0)

### Mediating effect of child ASF consumption and household food security on the relationship of livestock ownership with child anaemia

Analysis showed no evidence of mediation of the association of livestock ownership (total number of free-range poultry, sheep and goats, and pigs) with child anaemia by consumption of ASFs (Table 5). However, we did observe evidence that household food security mediated association between household pig ownership andchild anaemia (Coef (SE): 0.008 (0.04)).

**Table 5:** Direct, indirect, and total effects of household livestock ownership on child anemia assessing mediation by consumption of animal-source foods and household food security.

	Mediating variable: ASF consumption		
	Number of free-range poultry	Number of sheep and goats	Number of pigs
	Coefficient (SE)	Coefficient (SE)	Coefficient (SE)
Direct effect	0.006** (0.003)	0.017** (0.006)	0.021 (0.014)
Indirect effect	-0.0003 (0.0005)	-0.0001 (0.0004)	-0.00005 (0.0003)
Total effect	0.006** (0.003)	0.017*** (0.006)	0.020 (0.0139)
	Mediating variable: household food security		
Direct effect	0.005^**†**^ (0.003)	0.015** (0.006)	0.012 (0.014)
Indirect effect	0.0007 (0.0005)	0.002 (0.001)	0.008^**†**^ (0.04)
Total effect	0.006 **(0.002)	0.017**(0.006)	0.020 (0.013)

^**†**^*P*<0.10

**P<0.05

***P<0.001

Coefficients are standardized path coefficients from models using maximum-likelihood estimation with robust standard errors. The coefficient for the direct effect is the partial regression coefficient of the association of number of household livestock ownership with child anemia. The indirect effect of the number of household livestock ownership on child anemia is calculated as the product of the path coefficient between the number of household livestock and the diversity of ASF consumed by children or household food security. The total effect is the sum of the direct and indirect effects of number of livestock ownership on child anemia; Child anemia is modeled as a dichotomous variable defined as hemoglobin < 110 g/L.

## Discussion and conclusion

Nearly half of the children in this study were anaemic. The odds of anaemia were higher in households with a greater number of free-ranging chickens, even after controlling for other household socio-demographic characteristics. However, households with more livestock generally did not have children who consumed more ASFs, nor was household food security higher where children consumed more ASFs. Furthermore, household food security, intestinal parasitic infection, and children’s consumption of ASFs did not significantly mediate the relationship between household livestock ownership and child anaemia.

Although the presence of free-ranging poultry has been associated with childhood infections, particularly of *Salmonella* and *Campylobacter* species through faecal carriage in children [28, 29], our study found no association between the ownership or number of free-ranging chicken and intestinal parasitic infections (*Schistosoma haematobium*, *S*. *Campylobacter species)* in children. This link was our hypothesized pathway through which chickens might increase the risk of anaemia. While acknowledging the complex relationship between infections and childhood anaemia, the observation that free-range poultry ownership does not predict infection, but does predict anaemia, may be a result of the overall low prevalence of childhood infections in the sample. In addition, close to half (46.8%) of the study children had taken medication against intestinal and urinary tract parasitic worms within 12 months prior to data collection. Furthermore, a little more than a quarter of children (29%) reportedly received antibiotic treatment for fever and more than one-tenth (11.7%) for diarrhoea.

Conversely, the association between malaria and anaemia in the study children was clearly established. *Plasmodium* infection remained an independent significant predictor of anaemia. This finding is consistent with those of other studies demonstrating that children with malaria are more likely to be anaemic compared to their counterparts without *Plasmodium* parasites [30, 31]. Indeed, other investigations have identifyed malaria as a leading cause of anaemia in children in sub-Saharan African countries [32, 33]. Malaria parasites cause haemolysis of erythrocytes and bone marrow dyserythropoiesis. This compromises the formation of red blood cells and prevents quicker recovery from anaemia. A review of 29 community malaria intervention studies found that < 5-year-old children who received 1–2 years of malaria control had a 27% lower mean relative risk for mild (Hb< 11g/dL) anemia, and a 60% lower risk for more severe (< 8 g/dL) anemia, compared with their counterparts not exposed to any malaria interventions [34, 35].

The cross-sectional nature of our study design limited our ability to evaluate the mechanisms by which past intestinal parasitic infections and malaria influence anaemia. Such information would have helped to separate the contributions of homolyses due to past infections and asymptomatic low grade infection on haemoglobin [36]. The pathophysiology of malarial anaemia has long been established in numerous studies and can be summarized mainly as increased red blood cell destruction and/or decreased red blood cell production resulting in reported low haemoglobin level in infested persons [37, 38].

For intestinal parasitic infections, about half of the children had recently taken an anti-helminth drug, complicating the capacity of a single time point study to clarify this infection-anaemia relationship. In particular, we were unable to detect the effect of recently cleared infections on haematological recovery and current haemoglobin. In other words, previously cleared infections could have contributed to current childhood anaemia status which we could not test for using the diagnostic methods employed. Additionally, day-to-day variation in faecal egg output among soil-transmitted helminths produces variation in test results, even on consecutive days, limiting inferences based on single faecal samples [39].

Consistent with other published reports, household ownership of more livestock, particularly goats, sheep, and cattle, in our study was associated with improved household food security [40–42]. Ownership of livestock may reduce household food insecurity through increasing available disposable income that could be used to purchase food, thus increasing food access. It also might directly increase the availability of ASFs for home consumption, particularly, eggs from poultry.

To better evaluate the observed association between household poultry ownership and childhood anaemia, other biomedical tests, for example to determine serum hepcidin levels will be needed. As compared to haematocrit or haemoglobin, hepcidin represents a sensitive indicator of iron deficiency, even in the absence of anaemia. Reduced hepcidin is an early marker of transferrin saturation and decreased ferritin [43]. Hepcidin levels are also elevated following inflammatory conditions such as that produced by infections [44]. Thus, determination of hepcidin levels together with serum ferritin and transferrin would better diagnose the underlying pathway of childhood anaemia. In addition, faecal samples from the poultry could be collected to compare their pathogen DNA with that found in the stools of exposed children. This should help identify specific infections that were transmitted from poultry.

Given the complex and multi-faceted aetiology of anaemia, combating childhood anaemia calls for a multi-sectoral approach. Nutrition-sensitive agricultural activities, such as livestock production, have recently received attention as a possibly effective measure for improving household food security and the quality of diets. However, household ownership of livestock, particularly free-range poultry, may negatively affect haemoglobin levels of children living in those households. The findings from our study further highlight the need to promote household livestock production, but also and suggest that improved animal husbandry practices, especially corralling household livestock, could reduce unintended negative effect on children who are exposed to infectious agents from these animals.

## Supporting information

S1 File Dataset. (DTA)
